# Assessment of the culture of safety in public hospitals in Brazil^1^

**DOI:** 10.1590/1518-8345.1600.2849

**Published:** 2017-03-09

**Authors:** Rhanna Emanuela Fontenele Lima de Carvalho, Lidyane Parente Arruda, Nayanne Karen Pinheiro do Nascimento, Renata Lopes Sampaio, Maria Lígia Silva Nunes Cavalcante, Ana Carolina Pinto Costa

**Affiliations:** 2 PhD, Adjunct Professor, Centro de Ciências da Saúde, Universidade Estadual do Ceará, Fortaleza, CE, Brazil.; 3 Doctoral student, Universidade Estadual do Ceará, Fortaleza, CE, Brazil. Scholarship holder at Coordenação de Aperfeiçoamento de Pessoal de Nível Superior (CAPES).; 4 Undergraduate student in Nursing, Universidade Estadual do Ceará, Fortaleza, CE, Brazil. Scholarship holder from Fundação Cearense de Amparo à Pesquisa (FUNCAP), Brazil.; 5 Master's student, Universidade Estadual do Ceará, Fortaleza, CE, Brazil.; 6 Undergraduate student in Nursing, Universidade Estadual do Ceará, Fortaleza, CE, Brazil. Scholarship holder from Conselho Nacional de Desenvolvimento Científico e Tecnológico (CNPq), Brazil.

**Keywords:** Patient Safety, Organizational Culture, Health Personnel, Hospital

## Abstract

**Objective:**

to assess the culture of safety in three public hospitals.

**Method:**

transversal study undertaken in three Brazilian public hospitals, with health professionals through applying the Safety Attitudes Questionnaire (SAQ). Scores greater than or equal to 75 were considered positive.

**Results:**

a total of 573 professionals participated in the study, including nurse technicians and auxiliary nurses 292 (51%), nurses 105 (18.3%), physicians 59 (10.3%), and other professionals 117 (20.4%). The mean of the SAQ varied between 65 and 69 in the three hospitals. Among the domains, however, 'Job satisfaction' presented a higher score, and the opposite was observed for the domain 'Perceptions of management'. The outsourced professionals presented a better perception of the culture of safety than did the statutory professionals. The professionals with higher education presented a better perception of the stressing factors than did the professionals educated to senior high school level.

**Conclusion:**

the level of the culture of safety found is below the ideal. The managerial actions are considered the main contributing factor to the culture's weakness; however, the professionals demonstrated themselves to be satisfied with the work.

## Introduction

The culture of safety is defined as the product of values, attitudes, perceptions and skills, both group and individual, which determine a standard of behavior and commitment to safety in the institution, substituting blame and punishment with the opportunity to learn from failures^1^.

In Brazil, the topic became more evident in 2013, with the creation of the National Patient Safety Program (PSNP), under which the culture of safety was considered one of the principles for risk management geared towards quality and patient safety. Since then, the recognition of its importance and impact in health organizations has been the basis for undertaking any type of safety program, with emphasis on learning and organizational improvement.

The PNSP instituted, in Brazilian health establishments, the creation of the Nuclei for Patient Safety (NSP), with the aim of promoting and supporting initiatives directed towards patient safety. The nuclei are responsible for the development of a patient safety plan which identifies and describes strategies and actions defined by the Service for mitigating healthcare-associated incidents. One of the initial strategies of the NSP is to establish the culture of safety in the health institutions^2^.

However: prior to undertaking any action which promotes the culture of safety in the institution, the action must firstly be assessed and understood. One of the principal benefits of this assessment is to provide a concrete indicator of the current state of the culture, as well as to monitor its progression after the implementation of improvements. This evaluation may be undertaken rapidly and reliably through questionnaires, so as to obtain the maximum information regarding the organizational factors which influence issues of safety, and it is based on this understanding that the actions may be planned^3^.

Various instruments in various languages are available for evaluating the culture of safety. In Brazil, two instruments are being used for this assessment in hospital institutions^3^ ^-^ ^4^. For this study, the Safety Attitudes Questionnaire (SAQ) was chosen, in the version adapted and validated for the Portuguese language^3^ ^-^ ^5^. As well as being one of the instruments used most for assessing the culture of safety in various countries^6^ ^-^ ^8^, this instrument was chosen due to its practicality and the fact that it is possible to complete it rapidly, this process requiring approximately 10 minutes. Furthermore, its results can be associated with the patient safety indicators.

This study's hypothesis is that the in-depth assessment of the culture of safety allows a broader view of the indicators affecting patient safety in the hospital context. This entails the planning of actions geared towards quality care for the patient, with emphasis on organizational improvement.

Considering assessment to be the first step for establishing a safe culture, this study's objective was to assess the culture of safety of three hospitals in the State of Ceará (CE) through the use of the Safety Attitudes Questionnaire (SAQ).

## Method

A transversal study was undertaken in the clinical and surgical wards, outpatient centers, surgical centers, intensive care units and emergency units of three hospitals in the State of Ceará (CE), Brazil.

The study was financed by the Research Program for the SUS : shared management in health (*Programa Pesquisa para o SUS: gestão compartilhada em saúde* ) (PPSUS). A period of two years was established by the financing agencies for the undertaking of the study. In this way, the number of hospitals studied was determined by the time necessary for assessing the culture and hospitals with more than 300 bed spaces, defined in the literature as an approximate time of three months^(3)^. As a result, among the seven hospitals in the State which are centers of excellence, two were excluded due to having participated in the validation of the instrument used in the study, and the remaining five participated in a draw, in which three hospitals were chosen at random.

Hospital A is a center of excellence in mental health in the State of Ceará, hospital B is a center of excellence in attendance to persons with infectious-contagious diseases, and hospital C is a General Hospital which attends various medical specialities. All the hospitals are linked to universities and undertake teaching and research activities.

The professionals who participated in the study were divided into: nursing team (nurses, nurse technicians and auxiliary nurses) and workers from other categories (physician, physiotherapist, psychologist, social worker, nutritionist, occupational therapist and pharmacist).

In order to participate in the study, the health professionals had to work at least 20 hours per week and to have worked in the department for at least one month. Those professionals who were not undertaking work activities in the data collection period were excluded.

The professionals who accepted to participate in the study signed the Terms of Free and Informed Consent. The instrument was filled out in the work environment and was available on paper. In total, 950 instruments were distributed to the professionals from the three hospitals, approached randomly in their workplaces; however, 573 (60%) professionals returned the questionnaire completed.

The data were collected using the Safety Attitudes Questionnaire, validated and adapted for the context of Brazilian hospitals^3^. The instrument contains 41 questions which measure the professionals' perception regarding the culture of safety through six domains: Teamwork Climate, Safety Climate, Job Satisfaction, Stress Recognition, Perceptions of Management and Working Conditions. The response to each question follows a five point Likert scale. The final score varies from 0 to 100. Scores ≥ 75 are considered positive^5^.

After the data collection, the information from each question was inserted and processed using the Statistical Package for the Social Sciences (SPSS) version 18.0. The level of significance considered for the study was 0.05. The categorical variables were described in absolute and percentage numbers, and the quantitative variables were expressed by mean and standard deviation. In order to compare the means, the Fisher two-tailed test was applied for the categorical variables, and the Student t-test or Mann-Whitney test was applied for the ordinal quantitative variables.

The study was approved by the ethics committee of the three institutions, and was documented under protocol number 107,862. Anonymity was guaranteed to the study participants.

## Results

In the three hospitals, we obtained 573 completed instruments, representing a return rate of 60.3%, of which 106 (18.5%) were from Hospital A, 183 (31.9%) from Hospital B, and 284 (49.6%) from Hospital C.

In relation to the study subjects' characteristics, females predominated in all three hospitals, with a total of 434 (75.7%). Length of service varied for each hospital; however, in all hospitals, the largest percentage of professionals presented more than five years' service, it being the case that in Hospitals B and C, the largest percentage had between 11 and 20 years of service, while in hospital A, 32.4% of the professionals had length of service of 21 years or over.

The predominant system under which staff were employed was statutary, with 279 (48.7%) professionals - however, in Hospital C, the number of professionals who were outsourced and not statutary or who were employed on a temporary basis, such as the residents, had the highest percentage (54.9%).

Regarding professional category, the nurse technicians and auxiliary nurses were the professionals most likely to complete the instrument, with a total of 292 (51%), followed by nurses 105 (18.3%) and physicians 59 (10.3%).

### Descriptive analysis of the responses to the Safety Attitudes Questionnaire

The perception of the climate of safety varied according to hospital, sex, length of service, professional category, system under which staff were employed and the professional's educational level. In relation to the total score obtained, none of the three hospitals presented a positive value. However, among the domains, Job Satisfaction presented the highest score in all the hospitals, while the opposite was observed for the domain Perceptions of Management, which presented the lowest values.

Regarding the total value of the scores, and the values by domain, of the three hospitals, a similar value for the instrument's total mean was found in two hospitals, Hospital C having a mean of four points less, with a statistically significant difference. By domain, the mean varied between 57 and 80 for Hospital A, 58 to 75 for Hospital B, and 53 to 81 for Hospital C, with statistical differences in the domains of Safety Climate, Perceptions of Management and Working Conditions (Table 1).

**Table 1:** Distribution by domain, of the Safety Attitudes Questionnaire (SAQ), of the means of the three hospitals in the study. Fortaleza, CE, Brazil, 2015

Domains of the SAQ	Hospitals			
	A		B		C	
	Mean	SD	Mean	SD	Mean	SD
SAQ Safety Attitudes Questionnaire total	69	13	69*	13	65	13
Teamwork Climate	75	17	75	18	73	18
Safety Climate	65	19	67*	17	63	18
Job Satisfaction	80	17	83	18	81	16
Stress Recognition	76	25	74	25	73	27
Perceptions of Management						
Unit	60*	23	58	23	53	28
Hospital	61	25	64*	23	54	25
Working Conditions	57	25	66*	27	64	29

*p<0.05

In relation to length of service, the professionals with less than six months of service presented a better score in all the domains and in the total, in comparison with the other professionals whose length of service was superior to six months. A statistically significant difference was observed in relation to the comparison of the means in the domain Working Conditions for those professionals with less than six months (77). The professionals whose length of service was 21 years or over were those who presented the highest score in the domain Job Satisfaction (85). The domains of Perceptions of Management were those that presented the lowest mean with all the professionals (Table 2).

**Table 2:** Distribution of the mean by category and length of service, by domain and total score of the Safety Attitudes Questionnaire (SAQ). Fortaleza, CE, Brazil, 2015

Length of service	Safety Climate	Stress Recognition	Perceptions of Management:		Working Conditions	Teamwork Climate	Job Satisfaction	SAQ* total
			Of the hospital	Of the unit				
	Mean	Mean	Mean	Mean	Mean	Mean	Mean	Mean
Less than 6 months	67	73	63	64	77*	78	82	72
6 to 11 months	64	69	59	51	62	76	76	65
1 to 2 years	65	77	63	60	68	74	81	68
3 to 4 years	65	77	63	56	65	73	81	68
5 to 10 years	64	75	56	56	58	75	80	66
11 to 20 years	64	74	56	53	64	73	82	66
21 years or over	66	72	58	54	60	74	85	67

*p<0.05

In order to ascertain whether there was a difference in the perception of the Culture of safety in relation to the nature of employment with the institution, the means of the SAQ were compared with the type of employment. The outsourced staff presented, on average, a total score for the questionnaire which was greater than that of the statutary professionals, 69 and 66, respectively. This difference was considered to be statistically significant (p<0.05).

In relation to the domains, the outsourced professionals presented a difference in that their mean scores were higher than those of the statutary professionals in the domains: Perceptions of Management of the unit and Working Conditions; both with a statistically significant difference. The perception of stress was greater among professionals employed by the state, and the domains of Job Satisfaction and Teamwork Climate obtained the same value for the two types of employment (Table 3).

**Table 3:** Distribution of the mean by type of employment by domain of the Safety Attitudes Questionnaire (SAQ). Fortaleza, CE, Brazil, 2015

	Type of employment		
	Statutary		Outsourced		
	Mean	SD	Mean	SD
Safety Climate	63	18	66	17
Stress Recognition	75	26	74	27
Perceptions of Management - hospital	57	26	62	23
Perceptions of Management - unit	53	26	59*	26
Working Conditions	59	28	69*	26
Teamwork Climate	74	18	74	17
Job Satisfaction	82	18	82	15
SAQ total	66	14	69*	12

*p<0.05

Higher means were observed for the nursing team in the questionnaire's total score. In relation to the domains of Safety Climate, Perceptions of Management of the unit and of the hospital, and Working Conditions, this difference was statistically significant, when compared to the professionals of other categories. The opposite was observed in the domain Stress Recognition, in which the professionals from other categories presented a high-end mean in comparison with the nursing professionals. At this point, the domain of Stress Recognition presented the highest score among the professionals educated to degree high level, with a statistically significant difference (Table 4).

**Table 4:** Mean of the scores of the professionals educated to higher education level and senior high school level, and of the nursing team and of other categories. Fortaleza, CE, Brazil, 2015

	Higher education level		Senior high school level		Nursing team		Other professionals	
	Mean	SD	Mean	SD	Mean	SD	Mean	SD
Safety Climate	63	20	65	17	66*	17	60	21
Stress Recognition	81*	21	69	28	71	28	85*	16
Perceptions of Management - Hospital	61	25	56	25	59*	25	57	26
Perceptions of Management - Unit	53	24	57	27	57*	26	49	25
Working Conditions	62	25	64	30	65*	29	58	24
Teamwork Climate	75	18	73	17	74	17	75	19
Job Satisfaction	81	17	82	16	82	17,09	79	17
SAQ total	67	13	66	14	67	13,91	65	13

*p<0.05

## Discussion

The present study assessed the perception of the culture of safety of the professionals from three hospitals in the State of Ceará, through the use of the Safety Attitudes Questionnaire (SAQ). The SAQ is one of the instruments used most for assessing the culture of safety in health institutions^6^. This study presents the first quantitative assessment of the culture of safety, through the perception of the climate of safety, undertaken in the State of Ceará.

In Brazil, the professionals who make up the nursing team, due to being the majority of the professionals in the health institutions, were also those who most responded to the questionnaire. The physicians were those who most declined to answer the questionnaire, alleging lack of time for filling out the scale. This low frequency in the physicians' responses has also been observed in other studies with the FAQ, as well as in studies using other questionnaires with the same purpose^9^ ^-^ ^10^.

None of the three hospitals presented a positive general score, corroborating studies undertaken in other Brazilian states, which obtained total scores for the SAQ below 75. The State of Ceará obtained a score above that obtained in a study in a teaching hospital in Brasília^11^, but similar to that obtained in studies undertaken in public hospitals in the states of São Paulo and Minas Gerais^12^ ^-^ ^13^.

A statistically significant difference was identified in the comparison of the means of the hospitals' scores. Hospital C presented a general value for the SAQ which was below that of the other hospitals, reflecting the weakness of the culture of safety according to the professionals' perception. However, one explanation for the difference of the mean between the hospitals may be due to the fact that Hospital C is the largest, and the only General Hospital among the three hospitals evaluated, with different specialities.

The majority of the professionals are female, employed on a statutary basis and with length of service in the institution of more than five years. This profile corroborates that found in other studies assessing the culture of safety^12^ ^-^ ^14^.

The domain Job Satisfaction was the one which received the best result in the three hospitals. When compared with the mean observed in other Brazilian studies^(11-15)^, one can perceive positive scores, of over 75, demonstrating that on average the Brazilian professionals are satisfied with the work that they undertake.

In comparing this study with international baseline studies, one can infer that the Brazilian professionals are more satisfied with their work than are the professionals of other countries such as the United States of America (USA) and Ireland^(16-17)^. The Brazilian professionals' mean was below only the results in Switzerland^(18)^. A score above 80 means that there is a strong consensus among the professionals regarding the institution's climate of safety^(5)^. In other words, the institution has an environment which is propitious for the work and the professionals are satisfied with the work that they carry out, contributing to positive attitudes to safety.

The opposite was also observed; the domain of Perceptions of Management had the lowest score among the six domains. Studies undertaken in other Brazilian cities obtained similar results^12^ ^-^ ^15^, this was also the case in international studies undertaken in the USA, China and Ireland^16^ ^-^ ^17^ ^,^ ^19^.

Another domain which deserves to be emphasized is Stress Recognition, in which a score close to the positive value was presented (74.3). It stands out that the professionals educated to graduate level had greater recognition of the stressing factors which influence the undertaking of the work, with higher scores in comparison with the professionals educated to senior high school level. Professionals who participated in an assessment of the culture of safety in a public hospital in the Brazilian capital also demonstrated - by the positive score value - that they recognize when the stressing factors influence the undertaking of the work^11^.

A study held in Norway and Hungary shows results which are similar to the Brazilian studies, in which the professionals present a good perception of stressing factors in the work environment^20^ ^-^ ^21^. Occupational stress is commonly reported by health professionals, principally among professionals from the nursing team^22^. Studies have demonstrated that problems relating to workload and to restrictions on autonomy can result in emotional exhaustion and aversion to the patient^22^ ^-^ ^23^. Failure to deal with these stressors can result in work errors, reduction in productivity, feelings of discomfort, and illness or poor performance by the team. Stress management, therefore, is of great importance and relevance for patient safety^23^.

Regarding length of service, the professionals with less than six months' length of service presented better scores in 5 of the 6 domains, and in the total score, in comparison with the other professionals whose length of service was superior to six months.

This difference was equally observed in a Spanish study with the same domains of Job Satisfaction and Perceptions of Management^24^. The professionals with less than six months' service in the institution are still attempting to adapt to the work environment and sometimes perceive the institution positively. At this point, the older professionals managed to perceive better the individual and collective skills which determine the institution's commitment and style in relation to issues of safety.

Another point investigated in the study was the interest in knowing whether there was a difference or not between the perception of the culture of safety between the statutary and outsourced professionals. The outsourced staff presented, on average, a total score for the questionnaire which was higher than that of the statutary professionals, 69 and 66, respectively. This difference was considered statistically significant. Moreover, the perception of stress was greater among the state-employed professionals, and the domains of Job Satisfaction and Teamwork Climate obtained the same value for the two types of employment.

This result may be associated with the fact that the casually-employed professional, who does not have stability in the job, presents positive results regarding the culture of safety due to fearing retaliation in the work environment - in spite of the confidentiality of the data having been emphasized innumerable times during the study. It is believed that - due to this being a context which is highly specific to the workers in the area of health in the State of Ceará - studies were not identified which used the comparison of these variables for comparison with the results found in this research.

The nursing professionals present a better perception of the culture of safety in comparison with the other professionals. Regarding the domains of Safety Climate, Perceptions of Management of the unit and of the hospital and Working Conditions, this difference was statistically significant, when compared with the professionals of other categories. The opposite was observed in the domain Stress Recognition, in which the professionals of other categories presented a higher mean in comparison with the nursing professionals, showing that the professionals of other categories recognise better the stressing factors which influence the undertaking of the work than do the nursing professionals. One study undertaken in Norway compared the SAQ scores between physicians and nurses and identified a statistically significant difference in all the domains for nurses^20^.

However, another Brazilian study, undertaken in Minas Gerais, found that physicians had a better perception of the management and of the working conditions in comparison with the nursing team^12^.

The information on safety culture can guide the interventions in the search for quality in the health services. It may be observed that there is a growing interest on the part of health institutions in studies on the assessment of the Safety Culture, as this is considered the first stage for the construction of the patient safety commission established in Ministerial Ordinance 529 of 1^st^ April 2013, which instituted the National Patient Safety Program across Brazil^25^.

Finally, this was the first study on the evaluation of the culture of safety and health institutions in the Northeast of Brazil, which implies difficulty in comparison of results. This type of evaluation is a measurement over time of this construct and there is a need for an assessment with triangulation of methods for a more in-depth analysis.

It is valid to highlight that in any evaluation study, whether on the climate of safety or on the organizational climate, the results generated through the application of questionnaires cannot be interpreted in isolation. They must be evaluated in conjunction with the institution's organizational characteristics and values and mission. As a result, we understand that the SAQ can be used as one more managerial tool for decision-making, with the aim of planning and producing a favorable work environment which is propitious for the professionals' satisfaction and motivation, and which consequently ensures quality care for the patient. Thus, emphasis is placed on the importance of undertaking further studies on the assessment of the patient safety culture.

## Conclusion

The professionals do not present a strong agreement regarding the issues of patient safety in the institutions, although they present good satisfaction in the work. The values obtained in the domain Perceptions of Management present values below 60, indicating that there is no general approval of the actions of management in relation to issues of safety.

The professionals educated to graduate level present better perception of the stressor factors than do the professionals educated to senior high school level, and the outsourced professionals presented better means when compared with the professionals educated to graduate level; however, this difference may be attributed to a number of factors, such as poor stability of employment and fear of retaliation.

The nursing team presented higher scores than the other professionals, with the exception of Stress Recognition, in which the professionals seem not to recognize the stressing factors which influence the undertaking of the work.

The level of safety culture found is below the ideal. The managerial actions are considered to be the principal factor contributing to the weakness of the culture; however, the professionals demonstrated satisfaction with the work.

The scores below the satisfactory level represent a warning sign regarding which domains need to be worked on in the institutions. As a result, the investigation and discussion of the domains which concern the culture of safety, through the application of the SAQ, will contribute to the improvement of the health service provided.
